# Simulation Approaches Used for Management and Decision Making in the Beef Production Sector: A Systematic Review

**DOI:** 10.3390/ani14111632

**Published:** 2024-05-30

**Authors:** Tek Raj Awasthi, Ahsan Morshed, Thomas Williams, Dave L. Swain

**Affiliations:** 1 School of Health Medical and Applied Sciences, CQUniversity, Rockhampton North, QLD 4701, Australia; t.m.williams@cqu.edu.au (T.W.); dave.swain@terracipher.com (D.L.S.); 2 School of Engineering and Technology, CQUniversity, Melbourne, VIC 3000, Australia; a.morshed@cqu.edu.au; 3 TerraCipher, Alton Downs, QLD 4702, Australia

**Keywords:** simulation, modelling, decision making, systematic review, beef production, livestock systems

## Abstract

**Simple Summary:**

Simple Summary: The global demand for livestock products is increasing, and to meet this demand there is a need for more research efforts to increase livestock production. Despite varieties of simulation models developed and used in the beef production sector, an overview of available research is lacking. A systematic literature review was conducted to provide an overview of available beef production simulation modelling research and to describe simulation approaches used for livestock management. A growing research interest in simulating beef production systems was noted, with the main characteristics of the studies being biophysical and bioeconomic study types, deterministic and dynamic simulation approaches, whole-farm scenarios, and a focus area of productivity and economy. We recognized the need for improving the availability of information related to model validation techniques and type of software or programming languages used, which could facilitate the further research extension and/or adoption of simulation modelling studies in livestock management.

**Abstract:**

Simulation models are used in various areas of agriculture to better understand the system and assist in decision making. In the beef production sector, a variety of simulation research focusing on various dimensions of the system is available. However, an overview of the available research is lacking. Therefore, a systematic review was conducted to provide an overview of simulation studies of beef production and create an understanding of the simulation approaches used. Scopus, Web of Science, and ProQuest Central research databases were used to search the relevant articles, with the last search conducted in June 2023. Studies that developed or used simulation strategies and used beef cattle as a primary focus of the study were included. The 105 studies included in this review were examined thoroughly to record the authors, year of publication, country of study, type of study, focus area of the study, simulated scenarios, validation methods, and software programs used. There has been growing research interest in simulating beef production systems worldwide, with most studies conducted in North America and Europe. Among these studies, the majority (84.76%, *n* = 89) are biophysical or bioeconomic study types and use deterministic approaches (*n* = 42). Additionally, most studies have a whole-farm scope (38.09%, *n* = 40) and focus on productivity (51.43%, *n* = 54). Since only less than half of the studies mentioned the validation techniques and software programs used, there is a need to improve the availability of this information to ensure that the models are adopted effectively in decision making.

## 1. Introduction

Livestock production is a complex system that requires optimal decision making to bring about significant production improvements. Decisions made to implement any changes in livestock production could benefit from an analysis of risk and associated impacts [1]. Therefore, a detailed analysis of the possible impacts of the implementation of any changes is essential. To make informed decisions, we need to understand how the system works and the potential impacts of intervention to the production process. Simulation models, which provide the framework and tools to describe and understand the system [2], could be useful to evaluate the impacts of any decisions and hence help guide appropriate actions needed to improve the production. 

Mathematical equations have been used for more than a century to describe various components of animal systems. However, the development of greater capacity computing technologies has enabled the use of complex models to describe these systems in greater detail and to accurately predict future outcomes [3]. Recent advances in technology, mainly computing power and information technologies [4], provide an opportunity to use existing knowledge to develop novel strategies that help evaluate farm management and decision making [5]. 

Significant research has been undertaken in the simulation of livestock production systems, where the majority of studies have been conducted in crop–livestock [6,7,8] and dairy systems [9,10]. In addition to the crop–livestock and dairy industries, beef production is also highly researched. Since the beef production system around the world is very dynamic and heterogenous, the research available in simulating the system is also varied [11]. The availability of diverse simulation studies with a wide range of applications created a need for systematic documentation of available research to provide an overview of the studies and the strategies used to simulate the beef production system.

In this paper, we systematically review the available research on the simulation of beef production systems. The objectives of this review are to (1) provide an overview of simulation studies from the beef production sector, and (2) develop an understanding of strategies used to simulate the growth of an animal. To achieve our objectives, we propose finding answers to the following research questions: (1) What are the types of studies and approaches used in simulating beef production systems? And (2) what are the strategies used to simulate the growth of an animal?

## 2. Materials and Methods

This review adopts the statement of Preferred Reporting Items for Systematic reviews and Meta-Analyses (PRISMA) as a guide to identify, select, and synthesize the studies [12]. The checklists and revised flow diagram of the PRISMA 2020 statement were used to conduct and report this review.

### 2.1. Literature Search Strategy

An electronic database search was completed in June 2023 to obtain published literature that simulated beef production systems. Scopus, Web of Science, and ProQuest Central research databases were searched for relevant research articles. Search string (cattle OR beef) AND (model*) AND (simulation OR dynamic OR biophysical OR system) AND (“extensive beef production” OR “livestock production” OR cow-calf OR crop-livestock) AND NOT (dairy OR milk) was used with title, abstract, and keywords as search fields. A detailed bibliographic search for relevant articles was also performed to obtain potentially relevant articles otherwise missed during the preliminary database search. All the documents obtained from the database and bibliography search were exported to Microsoft Excel. Bibliographic details including authors, title of the document, year of publication, and abstract were recorded for all search results. Duplicates were automatically removed using Excel functionality, and a subsequent manual search of the record was also performed to find duplicates that were not removed during the automated process. A set of inclusion and exclusion criteria was set up to determine the relevance of the article to this review. Articles that met the following inclusion criteria were included in this study: (1) used beef cattle as a main focus of the study; (2) developed a simulation model or performed simulation experiment(s). Non-English articles were translated into English language using Google Translate. Literature reviews, working papers, dissertations, theses, and news articles were not included. Studies where simulation was performed but the strategies used for simulation were not described in detail were also excluded [13,14,15,16]. In other studies, where a previously developed strategy was either used to run simulations or modified to serve different purposes, an extensive search for the original paper was conducted through database and bibliography searches. If the original paper was found, eligibility was assessed, and the other papers referring to the original paper were excluded [17,18,19,20]. If the original publication was not found, the paper referring to the original paper was further explored to match the eligibility criteria [21].

### 2.2. Data Extraction and Analysis

A record of publication year, country of system studied, type of study, scope and focus area of the study, simulated scenarios, validation strategies, and software programs used was created in Microsoft Excel. The studies included in this review were categorized based on the system that was modelled and the simulation approaches used. Five categories of studies were created based on the system modelled: biological, biophysical, bioeconomic, agroecosystem, and population. If the study did not mention any of the categories mentioned above as the study type, the defining criteria set out in Table 1 were used to assign the study type. Additionally, the studies were categorized into seven types based on the simulation approaches used: static, dynamic, deterministic, stochastic, theoretical/conceptual, empirical, and other. The categorization of the study according to the simulation approach was performed only if the study specifically mentioned the approaches it used. Those studies that did not mention any approach were classified as using the “other” approach. For studies in which more than one simulation approach was used [22,23], all approaches were recorded.

The scope of each study was determined based on what level of beef production is represented. The studies were found to have five scope levels, as defined below:

1. Individual animal: studies that have an individual animal as a modelling unit and focus on the growth, reproduction, animal physiology, or management of an individual animal.
2. Cow–calf: studies that include the management of cow–calf operations, the interaction of cow and calf, or calving phenomena.
3. Herd: Studies that include a herd of animals as a modelling unit. For example, simulation of herd dynamics, herd management, or selection strategies.
4. Whole farm: Studies that include the whole-farm scenario. Such studies include animal, pasture, environment, and management strategies as input parameters.
5. Enterprise: studies that include components beyond the whole farm such as animal trade, farm-to-slaughter process, private or public entities, or government agencies.

The focus areas of the studies were diverse. For simplicity, seven main focus areas (productivity, economy, farm management, environment, animal health, pasture/forage, and resource use) were generalized based on the defining criteria in Table 2. The defining criteria for the focus areas were set based on the intended use of the studies. For example, if the study was intended to be used to evaluate or optimize production results [24], it was considered to have the focus area of productivity. Each study was found to have one or more focus areas. The model evaluation and validation strategies used in the studies were recorded as sensitivity analysis, comparison to available data, comparison to other studies, and face validity (expert panel review). Any strategies other than those mentioned above were recorded as “other”. Software or programs used to build the model or run simulations were also recorded.

Each study was examined for what scenarios were simulated. The scenarios simulated in the studies were diverse and classified as one or more of the nine scenarios tabulated in Table 3. The scenario was considered to be simulated if the study included the description or equations on how the variable associated with the scenario was computed or if the simulation experiment was conducted to obtain the value of the variable. If the study simply used the variable as model input or the data related to the variable were used to predict the value of a different variable, the scenario was not considered to be simulated. If the study was found to simulate animal growth, it was further explored to obtain the time step of growth simulation, stage of animal’s life simulated, and the purpose of growth simulation.

## 3. Results

### 3.1. Database and Bibliographic Search Results

**Figure 1:**
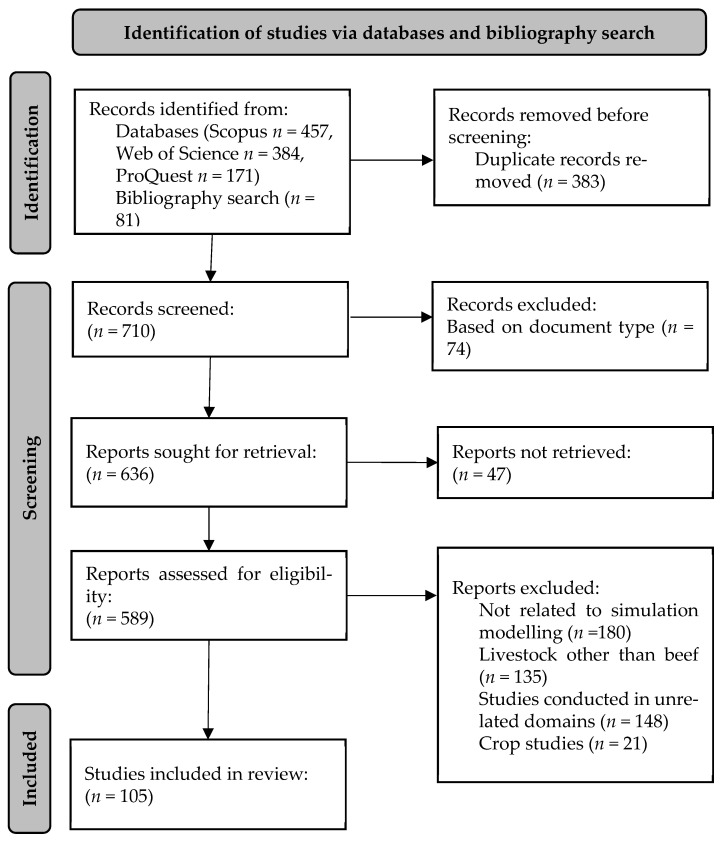
PRISMA diagram for identification of studies via databases and bibliographic search.

A total of 1012 documents were obtained from the database search of Scopus, Web of Science, and ProQuest. Of these, 37.85% (*n* = 383) were duplicates. The bibliographic search resulted in 81 unique and potentially relevant articles, which makes the number of unique documents examined to be 710. Approximately ten percent (*n* = 74) of unique documents were excluded based on their document types (e.g., review, working paper, thesis, news, etc.). Of the remaining articles (*n* = 636) that were sought for retrieval, 47 articles were not retrieved. Among the 589 articles that were accessed for eligibility, approximately 30.56% (*n* = 180) of the articles were excluded as they were not related to simulation modelling, a further 22.92% (*n* = 135) of the articles were related to livestock other than beef, such as dairy cows, pigs, sheep, or goats, and 3.56% (*n* = 21) of the articles were of crop studies. Some studies (25.12%, *n* = 148) were excluded because they were conducted in unrelated domains, such as epidemiology, forest management, marketing, etc. Combined from the database and bibliographic search, a total of 105 articles met the selection criteria to be included in this review (Figure 1).

### 3.2. Geographic and Year-Wise Distribution of Studies

**Figure 2:**
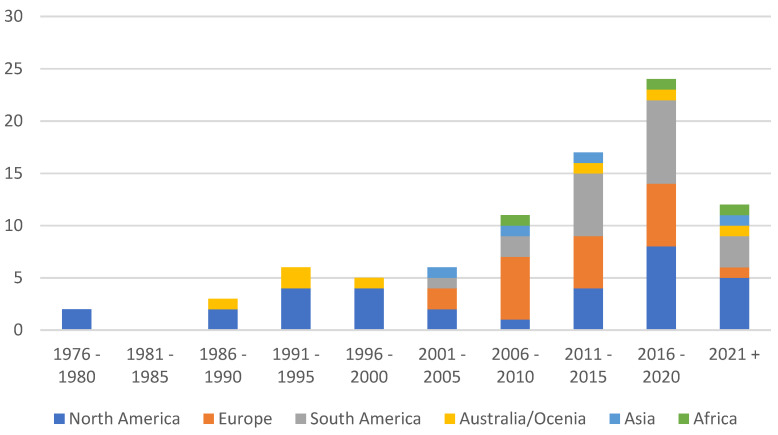
The number of studies identified for each year of database search based on the continent of the study.

The studies were conducted in 25 countries across six continents. Of the studies, 92.38% were conducted in North America (*n* = 38), Europe (*n* = 20), South America (*n* = 26), or Australia (*n* = 13). Only 7.62% of the studies were reported from Asia (*n* = 4) and Africa (*n* = 4). The country with the highest number of studies was the United States (*n* = 30), followed by Brazil (*n* = 17) and Australia (*n* = 11). The United States, Brazil, and Australia represented more than half of the studies. The studies were published between 1977 and 2023. Figure 2 shows the year-wise distribution of the studies with the number of studies conducted in each continent. The publication dates were grouped into 5-year periods to provide a more general assessment of the time patterns. Most studies were published after 1990 (*n* = 101), with approximately 83.81% (*n* = 88) of the studies published after 2000.

### 3.3. Overview of Simulation Studies

The overview of studies included in this review and their main characteristics and uses is presented in Appendix A (Table A1).

#### 3.3.1. Types of Studies and Simulation Approaches

**Figure 3:**
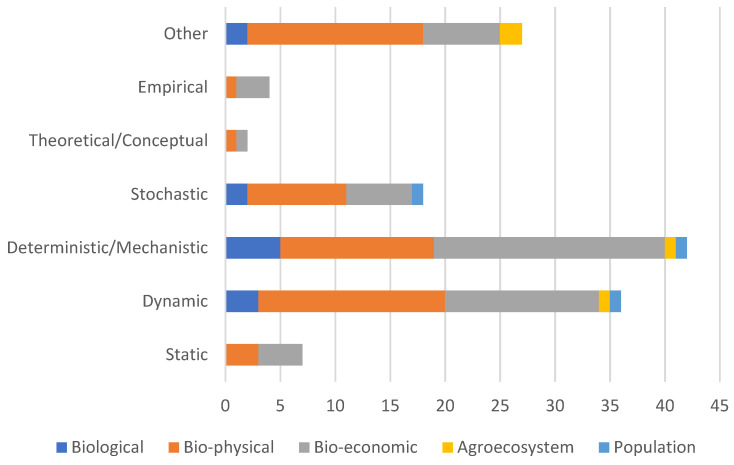
Number of studies based on system modelled and modelling approaches used.

Most of the studies were biophysical (*n* = 47) or bioeconomic (*n* = 42) studies. Biological (*n* = 9), agroecosystem (*n* = 4), and population (*n* = 3) studies all together accounted for only 15.24% of the studies. Single or multiple simulation approaches were used in the studies. Most studies used deterministic approaches (*n* = 42), followed by a dynamic (*n* = 36) and a stochastic simulation approach (*n* = 18). Only two studies used a conceptual approach. Of the total studies, 25.71% (*n* = 27) were found not to mention the approach used to simulate the system, in which case the approach was recorded as “other” (Figure 3).

#### 3.3.2. Scope and Focus Areas of the Studies

**Figure 4:**
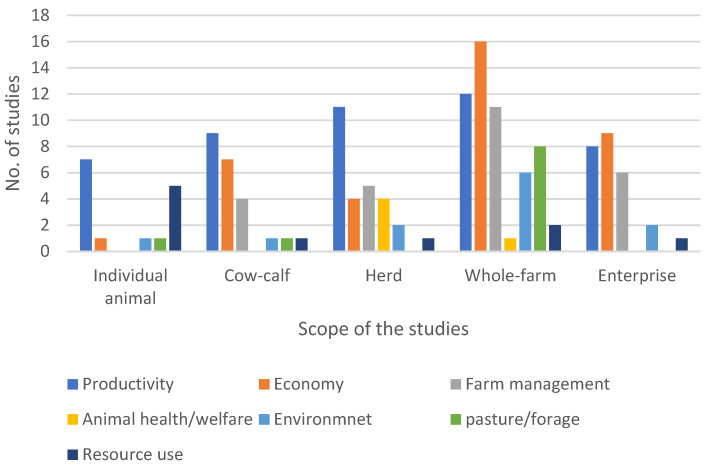
Number of studies based on the scopes and the focus areas of the studies.

The scope of the studies was categorized as individual animal, cow–calf, herd, whole farm, and enterprise. The majority of studies were categorized as whole farm (*n* = 40, 38.10%), followed by herd (*n* = 20, 19.05%) and enterprise (*n* = 18, 17.14%), whereas 11.43% (*n* = 12) and 14.29% (*n* = 15) of the studies were found to have a scope of individual animal and cow–calf, respectively. The studies were found to have one or more focus areas. Productivity (*n* = 54) and economy (*n* = 42) were the top major focus areas, followed by farm management (*n* = 27) and pasture or forage (*n* = 14). A total of 28 studies were focused on environment, animal health, and resource use combined. Among the studies with individual animal, cow–calf, and herd scopes, most studies were focused on productivity. However, for whole-farm and enterprise-level studies, economy was the greatest focus area. No studies of individual animal, cow–calf, and enterprise scope were focused on animal health and welfare. Similarly, no studies of herd and enterprise scope were focused on pasture/forage. There was at least one study from all scope levels that was focused on productivity, economy, and resource use (Figure 4).

#### 3.3.3. Validation Methods

**Figure 5:**
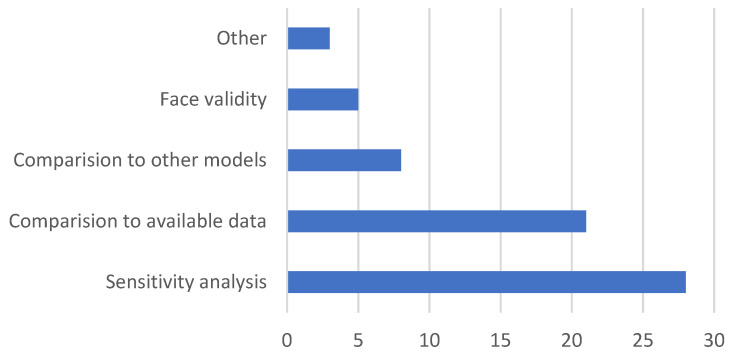
Number of studies with the type of validation techniques used.

Out of the total number of studies, only 49 (46.67%) mentioned model evaluation or validation techniques in their articles. Among these, sensitivity analysis (*n* = 28) was the most used technique, followed by comparison to available data (*n* = 21) and comparison to other studies (*n* = 8). The least number of studies (*n* = 5) adopted the face validity techniques. Additionally, a few studies (*n* = 3) utilized other validation techniques (Figure 5).

#### 3.3.4. Software and Programming Languages Used

Several computer software and programming languages were used in the studies. Only 48 (45.71%) of the studies included in this review mentioned the type of software or programming languages used to develop simulation models or conduct simulation experiments. Microsoft Excel (https://www.microsoft.com/en-au/microsoft-365/excel, accessed on 27 May 2024) was used in most studies (*n* = 16), followed by STELLA (https://www.iseesystems.com/store/products/stella-online.aspx, accessed on 27 May 2024) (*n* = 9). Programming languages R (https://www.r-project.org/, accessed on 27 May 2024) and FORTRAN (https://fortran-lang.org/, accessed on 27 May 2024) were used in 6 and 4 studies, respectively, whereas Java (https://www.java.com/, accessed on 27 May 2024), Visual Basic (https://dotnet.microsoft.com/, accessed on 27 May 2024), and C++ (https://isocpp.org/, accessed on 27 May 2024) were used in 3 studies each. Various other software and programming languages were found to be used, such as Oracle (https://www.oracle.com/, accessed on 27 May 2024), Model Maker (https://www.apbenson.com/about-modelmaker, accessed on 27 May 2024), Vensim (https://vensim.com/software/, accessed on 27 May 2024), Exsys (http://www.exsys.com/productmain.html, accessed on 27 May 2024), Zplan (https://bio.tools/zplan, accessed on 27 May 2024), MATLAB (https://mathworks.com/products/matlab.html, accessed on 27 May 2024), VISSIM (https://www.ptvgroup.com/en/products/ptv-vissim, accessed on 27 May 2024), SAS (https://www.sas.com/en_au/software/viya.html, accessed on 27 May 2024), @Risk (https://lumivero.com/products/at-risk/, accessed on 27 May 2024), Powersim studio 8 (https://powersim.com/powersim-studio/, accessed on 27 May 2024), and Lotus 1-2-3 (https://winworldpc.com/product/lotus-1-2-3/1x, accessed on 27 May 2024), and each of them was used in one study.

#### 3.3.5. Simulated Scenarios and Growth Simulation

Herd structure (*n* = 50), economic efficacy (*n* = 46), and nutrient requirements (*n* = 36) are the most frequently simulated scenarios in beef production studies. Other scenarios, such as forage/pasture, reproductive, emissions, resource use, animal health and disease, and genetic characteristics, were simulated in 28, 18, 12, 7, 6, and 4 studies, respectively (Table 3). Growth simulation was performed in only 27 (25.71%) of the studies included in this review. It was found that most studies (*n* = 12) used daily time step (determining the weight of an animal on a daily basis) to simulate the animal’s growth, whereas some studies used monthly time steps, and other studies used equations to predict weights at one or more stages of the animal’s life. Growth was simulated in each study either to produce animals’ weight as a model output or as an input to another component of the model. In each case, they have a specific purpose, as outlined in Table 4.

## 4. Discussion

### 4.1. General Trend of the Research

The studies included in this review were distributed disproportionately geographically, with North America and Europe as leaders in this area of research. The relatively large number of studies conducted in North America and Europe may reflect the large proportion of worldwide beef production in these regions [51] and the increasing interest in sustainable livestock production in developed countries. Similarly, South American countries such as Brazil and Argentina are among the top ten beef-producing countries [52], justifying the higher number of studies obtained from South America. However, the quantity of simulation research on beef production was much less for the Asian and African regions. Although China and India are on the list of the top five countries for beef production [52], the number of studies obtained from Asia is relatively low. One of the reasons for obtaining a small number of studies from Asia and Africa could be due to the lack of research funding in these regions. Acharya and Pathak [53] highlighted that, as of 2019, central Asia has the least share (0.1%) of global research spending followed by sub-Saharan Africa (0.8%). Some studies that are not included in this review have shown that there is a rapid increase in demand for livestock products in developing regions of the world [54,55,56]. Therefore, there is a need for more research towards adopting novel livestock management and sustainable production strategies in the developing parts of the world. It is also seen that there is growing research interest in the simulation modelling of beef production systems which is evident from comparing the number of studies published before 2000 (*n* = 17) and after 2000 (*n* = 88). The rapid increase in livestock research after 2000 was also highlighted by Escarcha et al. [11].

### 4.2. Overview of the Simulation Studies

Our review indicates that more than two thirds of the studies simulated biophysical or bioeconomic systems. However, approximately 59% of the studies did not mention the system they simulated. When considering these results, the use of defining criteria set out in Table 1 for the studies where the system is not specifically mentioned should be noted as a potential limitation. The removal of articles in which the system was not mentioned in the paper still shows biophysical (*n* = 10) and bioeconomic (*n* = 24) systems as the main simulated system compared to biological (*n* = 4), population (*n* = 3), and agroecosystem (*n* = 2). Thus, the high interest in biophysical and bioeconomic modelling is likely correct. The high number of biophysical and bioeconomic studies potentially reflects that studying the interaction between animal and physical components of the environment is important to understanding the complexity of livestock production. Biophysical and bioeconomic studies can provide a better representation of the real-life livestock production scenario compared to biological and population studies. Most biological studies are limited to simulating animal reproduction [57,58,59] or energy requirements [41,60], making them narrow focus studies. Although biological studies may provide an in-depth description of biological processes, they fall behind to include other aspects of livestock systems. Similarly, population studies [61,62,63] focus on population dynamics and provide less emphasis on the other important aspects of livestock production such as productivity or economic outcomes. Livestock production represents a complex phenomenon, and to understand how complex systems function, combining biological, physical, and economic aspects of the system is useful [64]. Therefore, biophysical and bioeconomic studies could provide a better representation of the complexity associated with livestock production systems.

It is observed that more than two thirds of the studies used deterministic or dynamic approaches. Another common approach was the stochastic approach. Static, empirical, and theoretical approaches were used less frequently. The simulation approaches can generally be divided into three categories: static vs. dynamic, deterministic vs. stochastic, and empirical vs. conceptual. Static approach represents the system at a specific point or duration in time. For example, a study by Foley et al. [65] represents adopting a static approach, which estimates GHG emissions in a single year without taking into account how the emissions change over the years. Similarly, a biophysical study by Kamilaris et al. [35] adopts a static approach to represent a farm system within a single year. A dynamic approach represents the changing nature of a system with an emphasis on how the system responds to different inputs over time. The Northern Australia Beef System Analyser (NABSA) [27], for example, simulates the dynamic nature of beef enterprise to observe the change in herd structure over time. Many dynamic studies [66,67,68,69] simulate how the herd structure changes over time, leading to productivity and economic risks or benefits. Simulation of pasture conditions [70,71,72] is also another major focus of dynamic studies. Among the studies reviewed here, one study [73] exceptionally used both static and dynamic approaches in a model; however, both approaches serve different purposes. The static approach was used to estimate the number of animals needed to maintain the required herd size, while the dynamic approach was used to predict the economic benefits of pasture improvements over time.

Deterministic and stochastic approaches can be differentiated by whether they consider uncertainties or randomness in the model output or not. Deterministic approaches tend to provide predictions based on the model inputs without considering the variability that could arise due to known or unknown factors. The study of forage intake in beef cattle [74] is a good example of a study using a deterministic approach, which predicts the forage intake by grazing cattle based on the sward characteristics. They simulated grazing behaviors (bite size, rate of biting, rate of intake, and grazing time) to determine the impact of forage quality and quantity on forage intake by cattle. Similarly, Gillard and Monypenny [34] deterministically determined the effect of drought and stocking rate on cattle properties in northern Australia. Stochastic approaches, however, incorporate the probability distribution of input parameters or the desired outcome representing the variability that is present in the system or might occur as a result of simulation. For instance, studies such as [21,63,75] used the stochastic nature of various parameters in the simulation model. Lancaster and Larson [21] used the energy required for maintenance, reproductive efficacy, nutritive value of forage, and forage yield as stochastic parameters, whereas Viet et al. [63] and Merico et al. [75] used the probability of occurrence of various reproductive and management events as model parameters. In contrast to studies employing either deterministic or stochastic approaches, some studies [22,48,76] use both approaches. Such studies usually contain sub-models with independent sets of parameters or provide independent outputs. 

Empirical and conceptual modelling approaches can be defined as either data-driven or hypothesis-driven approaches. Empirical studies are based on real-world observations and are supported by experimental data [77]. Studies by Fraiser and Pfeiffer [78] and Melton et al. [79] provide empirical examples to help make optimal decisions regarding herd management and breed choices, respectively. However, conceptual studies [46,80] are based on concepts and hypotheses related to the system.

Most biophysical studies used a dynamic approach, and most bioeconomic studies used a deterministic approach, which is evident in Figure 3. The common occurrence of biophysical simulators in agriculture was noted during the late 1990s by Oriade et al. [81], where they also pointed out that the use of dynamic approach in biophysical systems increases the accuracy of phenotypic prediction. However, the use of deterministic approach in bioeconomic studies can be linked to the potential use of the model. As most bioeconomic studies are used to assess/estimate the economic outcomes, accurate predictions are useful to make management decisions. As biophysical and bioeconomic studies share a common biological component, they also overlap in the use of modelling approaches. We observed some biophysical [23,49,82] and bioeconomic [66,83,84] studies adopting both dynamic and deterministic approaches. 

Most of the reviewed studies included the whole farm as a modelling unit with the majority of studies focusing on productivity and economy. Productivity and economy were the top focus areas, as most models have the objective of increasing production and hence improving the profitability of livestock production. Most of the studies with farm management as a focus area belong to the whole-farm scope, while no individual animal models were found to focus on farm management. This can be justified by the fact that livestock farms are complex systems and need interaction between various biotic and abiotic components, which could not be included in the models with the individual animal as a modelling unit. However, most individual animal models have the focus area of productivity, with potential use for assessing/optimizing production outcomes.

Among the validation techniques used in the studies’ sensitivity analysis was the main method, which is also the most often recommended method [85]. Sensitivity analysis is most commonly used to evaluate the impact of input parameters (that may have some degree of uncertainty) on the simulation output. Fort et al. [86], for example, performed sensitivity analysis of parameters that have greater uncertainty, where they used parameter values 20% below and above the normal range to observe the magnitude of variation it brings to the output. Similarly, Gradiz et al. [87] and Johnston et al. [88] performed sensitivity analysis to assess the impacts of changing values of input variables on the respective model outputs. Another common technique of model evaluation was the comparison of simulation results with the available data, such as adopted by Diaz-Solis et al. [89] and Ash et al. [27]. Diaz-Solis et al. [89] compared the prediction results with the field data collected from the experimental sites, whereas Ash et al. [27] used the regional herd data developed by the Cooperative Research Centre for Beef Genetic Technologies (CRCBGT) for comparison. Ash et al. [27] also compared the simulation results with previous studies such as Holmes et al. [90] and McGowan et al. [91]. A similar validation strategy where the simulation results are compared with the results of previous studies is also adopted in other studies [83,84,92]. The least common method of validation is face validity (*n* = 5), which involves the use of an expert panel review to determine how well the model represents the production system in real life and/or determine whether the outputs are reasonable given the expert’s experiences and expectations. Face validation can be used if a robust dataset or similar studies are not available for a direct comparison [35] or can also be used to provide an extra level of validation [25]. 

Among the computer software and programming languages, Microsoft Excel is the most commonly used software. The more frequent use of Microsoft Excel can be attributed to the ease of use and easy availability of the software. Although many studies use Microsoft Excel as the only software, its use is complemented often by the use of programming languages such as Java [93] and STELLA [89] to develop a user-friendly simulation interface. Among the other programming languages, R, FORTRAN, and C++ are most commonly used. FORTRAN is used frequently in earlier studies, with the latest study to use it being Thornton et al. [94] among the studies reviewed here. On the other hand, the R programming language is being used increasingly in recent studies. The increasing popularity of the R programming language after 2010 was also noted by Lai et al. [95]. This shows that there is a change in the popularity of the programming languages used in livestock simulation over a period of time. Since only 48 out of 105 studies mentioned which software and/or programming languages were used for simulation, the availability of information on the type of software programs needs to be improved. Providing information on software and programming languages is beneficial for its adoption and future development by researchers [96]. 

### 4.3. Simulated Scenarios and Growth Simulation

Our review indicates that herd structure, economic efficacy, and nutrient requirements are the most frequently simulated scenarios in the studies. Most of the reviewed studies have whole-farm scope with productivity and economy as a major focus, which explains the most frequent simulation of scenarios such as herd structure and economic efficacy. Most studies [24,26,40,97] that focus on increasing farm productivity include herd size and number of animals as an important indicator of productivity. Similarly, various studies [68,76,98] also make economic predictions based on herd structure. Therefore, the size and structure of the herd can be considered an important component in determining the productivity and economic returns of the farm. 

Less than one third (*n* = 27) of studies were found to simulate animal growth at any stage of the animal’s life. As we set up specific criteria to decide whether the scenario is considered to be simulated or not, as described in the Materials and Methods section of this paper, a low number of studies were obtained that met the criteria. We observed two distinct strategies employed to simulate the growth of an animal: one using nonlinear growth functions and another based on the metabolic energy available from feed. Most studies used nonlinear growth curve models such as Brody’s equation [32,36,42,48], Gompertz’s equation [29,33], and Richard’s function [40] to estimate the weight and growth. Such studies generally use the pre-existing data to fit the models and predict the growth patterns of animals. Another set of studies [22,28,31,47,49] is based on the concept that the energy available in the feed is firstly used for body maintenance and surplus energy is used for growth. Many of these studies adopt the net energy requirement equations developed in studies such as Fox et al. [99] and National Research [41]. Studies that implement the previously developed equations also tend to modify the equations to fit their unique scenario; however, the assumptions made to modify the equations are not always validated or are not well detailed. This may create confusion among model users on whether the model can be implemented in their unique production system or not. Our review indicates that only 47% of the studies explicitly mentioned the techniques they used to validate the model. The lack of detailed information on validation methods raises concerns about the trustworthiness of the model and potential adoption in livestock production. This may also cause doubt in adopting the model for further improvement or modifications to use in a different scenario. Therefore, future research in the livestock simulation sector should consider providing details on how the assumptions made in the study are validated and what the implications of adopting the model in a different scenario are.

## 5. Limitations

It would be useful to mention the limitations and delimitations of this review so that future studies could address them, and the current findings are interpreted correctly. Firstly, some articles might have used different terms to refer to simulation modelling, causing the exclusion of those articles in the database search using the search terms used in this study. Secondly, the simulation approaches and validation methods that are not frequently used in the studies are considered as “other” methods to simplify the classification and to make a clear analytical understanding of major simulation approaches and validation methods. This, however, does not mean that the approaches and methods noted as “other” are less important. Finally, this study reviewed the documents that have been published as journal papers, books, or conference proceedings, excluding other sources such as reports, working papers, and technical documentation that are not available online. As this review aimed to generate an understanding of simulation approaches used to simulate the beef systems, the models for which documentation detailing the simulation strategies was not available were not reviewed. This might have excluded the simulation models that are available as software or applications with no or limited information about the underlying mechanism of model functioning. However, we believe that this study generates significant knowledge by providing an overview of available research in simulation modelling of the beef sector and the need for further research in this area of knowledge.

## 6. Conclusions

This review provides an overview of 105 simulation modelling studies of beef production, highlighting the broad application of such studies in various dimensions of beef production. Studies were obtained from all over the world, with most of the studies conducted in North America. More than 80% of the studies were published after 2000, reflecting the growing research interest in this area. However, countries in the Asian and African regions still lack research efforts in livestock modelling despite the rapid increase in demand for livestock products in those regions. Our study found that biophysical and bioeconomic were the top study types with deterministic and dynamic simulation approaches as major approaches. Most of the studies simulated the whole farm scenario with a major focus on productivity and economy. Since only less than half of the studies mentioned the validation techniques and software programs they used, further studies should consider making this information available to facilitate effective adoption of the study in decision making.

## Figures and Tables

**Table 1:** Defining criteria set out for the type of system modelled in the study.

System	Criteria
Biological	Study focused only on simulating the biological processes of an animal, such as the reproductive process, animal physiology, growth rate, life cycle assessment, etc.
Biophysical	Study that includes biological (animal component) as well as physical components of a farm or environment. For example, studies that simulate the growth of cattle and also include farm characteristics, environmental factors, and other physical factors related to animals, farms, or the environment.
Bioeconomic	Study that includes economic or financial parts in addition to biological or biophysical components. Such studies tend to evaluate the economic risks of management interventions or predict the financial outcomes. They also include studies determining economic values of certain animal traits, cost analysis, income and expenses, development or evaluation of strategies to improve economic efficacy, etc.
Agroecosystem	Study that integrates livestock production with ecological modelling. Such studies may evaluate ecological sustainability of the livestock production or simulate the impacts of agricultural production on the ecosystem, such as studies related to grazing impacts.
Population	Study related to the simulation of cattle population dynamics. These studies are either related to cattle demographics or the impact of environment or management decisions on cattle population.

**Table 2:** Focus areas and their defining criteria.

Focus Area	Intended Use
Productivity	To assess/optimize production outcomes
Economy	To assess/estimate economic outcomes/financial performance
Farm management	To assist decision making/to evaluate management strategy
Environment	To estimate environmental outcomes or impacts
Animal health	To manage animal health and welfare
Pasture/Forage	To manage pasture/to assess or estimate forage production
Resource use	To assess/estimate/optimize resource requirement/use

**Table 3:** Scenarios simulated in the studies with the examples of simulated variables.

Simulated Scenario	Examples of Variables	No. of Studies
Herd structure	Herd size, herd dynamics	50
Economic efficacy	Economic values, various cost estimation	46
Nutrient requirement	Feed requirement, forage/pasture utilization, supplement requirement, metabolic energy requirement	36
Animal growth	Growth rate, liveweight, liveweight change, weights at different stages of animal life (birth weight, weaning weight, weight at calving, mature weight, etc.)	27
Forage/Pasture	Forage production, pasture production, pasture availability, forage quality and quantity	28
Reproductive	Gestation period, calving ease, estrous cycles and conception, calving rate	18
Emission	Methane, CO_2_, nitrogen, other GHG emissions	12
Health and disease	Animal health, animal disease, mortality, morbidity	6
Resource use	Land use, water consumption	7
Genetic characteristics	Genetic selection, genetic traits, genetic potential	4

**Table 4:** Growth simulation strategies used in the studies.

**Publication**	**Time Step of Growth Simulation**	**Stage/s of Animal Life Simulated**	**Purpose of Growth Simulation**
[25]	Daily	Birth to mature weight	Determine genetic potential, determine weaning weight, and mature weight
[26]	Daily	Post-weaning	Determine post-weaning daily gain
[27]	Monthly	Birth to mature weight	Determine productivity
[28]	Daily	n/a	Determine daily liveweight change
[29]	Daily	Birth to maturity	Estimate energy requirement from birth to slaughter
[30]	Daily	n/a	Estimate daily liveweight change
[31]	n/a	Maturity and pregnancy	Estimate weight gain and energy requirements
[32]	Daily	n/a	Estimate daily liveweight change
[33]	n/a	3 years to maturity	Estimate body weight and composition
[34]	n/a	n/a	Calculate weight of steers at sale
[35]	Monthly	n/a	Determine liveweights
[36]	n/a	Birth to maturity	Estimate liveweight
[22]	Daily	n/a	To estimate effect of growth on economic output
[37]	n/a	n/a	To estimate liveweight
[38]	n/a	n/a	Predict the potential liveweight gain based on the pasture availability and quality of pasture.
[39]	n/a	Birth to maturity	To determine the stages of animal such as birth, weaning, etc.
[40]	n/a	Birth to maturity	Estimate liveweight for forage intake estimation
[41]	n/a	Birth to maturity	To estimate energy and protein requirement for growth
[42]	Monthly	n/a	To calculate energy requirement
[43]	n/a	Birth to maturity	To calculate birth weight, weaning weight, and mature weight
[44]	Daily	Birth to calving	To determine animals’ liveweight at weaning, conception, and calving
[45]	Monthly	Birth to maturity	Use as input for GHG emission sub-model
[46]	n/a	n/a	Predict animal weight gain
[47]	Daily	Birth to maturity	Estimate growth rate
[48]	Daily	Birth to maturity	Determine animal performance
[49]	Daily	n/a	To determine animal production and forage utilization
[50]	Daily	During calving duration	To calculate postpartum anestrus

## Data Availability

The data utilized in this study have been extracted from the articles listed in Appendix A (Table A1) and are available from the corresponding author upon reasonable request.

## Appendix A

**Table A1:** Studies included in this review with their main characteristics and uses.

Publication	Country	Type of Study	Simulation Approach(es)	Scope	Focus Area(s)	Simulated Scenario(s)	Validation Method(s)	Software(s) Used
[100]	Norway	Bioeconomic	Deterministic	Cow–calf	Productivity, economy	Economic output	n/a	n/a
[25]	United States	Biophysical	Stochastic	Animal	Productivity	Nutritional requirement, animal growth, animal health, reproductive status	Face validity, comparison to available data	R
[26]	Italy	Bioeconomic	Deterministic	Enterprise	Economic and biological values	Herd composition, economic output, animal growth, nutrient intake	n/a	n/a
[101]	United States	Biophysical	Static	Cow–calf	Animal production	Water consumption	n/a	Microsoft Excel
[27]	Australia	Biophysical	Dynamic	Whole farm	Animal production, economy	Animal growth, mortality, economic output, herd dynamics, land condition, methane production	Comparison to other models	Microsoft Excel
[102]	Brazil	Biological	Other	Animal	Resource use	Feed intake	Comparison with available data	
[57]	United States	Biological	Stochastic, dynamic	Animal	Animal reproduction	Reproductive performance	Comparison to other models	n/a
[74]	United States	Biophysical	Deterministic	Animal	Resource use	Nutrient requirements	Sensitivity analysis, comparison to other models	n/a
[103]	Brazil	Biophysical	Deterministic, static	Herd	Productivity	Animal production, herd dynamics	n/a	n/a
[58]	France	Biological	Deterministic, stochastic	Animal	Animal reproduction	Reproductive output	Sensitivity analysis, comparison to available data	ModelMaker
[80]	United States	Biophysical	Theoretical/conceptual	Whole farm	Economy, farm management	Herd dynamics, reproductive output	Sensitivity analysis, comparison to available data	n/a
[104]	Brazil	Bioeconomic	Other	Herd	Economic, animal health	Herd dynamics, economic output	n/a	n/a
[66]	Brazil	Bioeconomic	Dynamic, deterministic	Cow–calf	Economy, productivity	Herd structure, economic output	n/a	Microsoft Excel
[28]	Uruguay	Biophysical	Other	Animal	Pasture, animal production	Forage production, animal growth	n/a	n/a
[105]	Brazil	Bioeconomic	Deterministic, static	Enterprise	Economy, productivity	Herd structure, economic output	n/a	Microsoft Excel
[106]	Chile	Bioeconomic	Stochastic	Cow–calf	Animal production, economy	Nutrient requirements, economic output, herd structure	Comparison to available data	Visual Basic
[29]	France	Biophysical	Other	Animal	Animal production	Animal growth, nutrient requirements	n/a	n/a
[107]	New Zealand	Bioeconomic	Deterministic	Whole farm	Management,	Economic output, environmental risks	n/a	n/a
[108]	Mexico	Agroecosystem	Other	Cow–calf	Animal production, forage	Forage production, nutrient requirements	Sensitivity analysis	STELLA
[89]	Mexico	Biophysical	Deterministic	Herd	Management, productivity	Herd dynamics	Comparison with available data	Microsoft Excel, STELLA
[70]	Canada	Biophysical	Dynamic, deterministic	Whole farm	Productivity, pasture	Pasture production, animal performance	Comparison with available data	STELLA
[82]	Australia	Biophysical	Dynamic, mechanistic, deterministic	Animal	GHG, feed intake	Methane production, feed intake	Comparison with available data, statistical	VISSIM, C++
[109]	United States	Biophysical	Other	Enterprise	Productivity, economy	Forage intake, forage production	Sensitivity analysis, comparison to available data	n/a
[110]	Brazil	Bioeconomic	Deterministic	Cow–calf	Economy	Economic output	Sensitivity analysis	Microsoft Excel
[65]	Ireland	Biophysical	Static	Whole farm	Environment	GHG emission	n/a	Microsoft Excel
[86]	Uruguay	Agroecosystem	Other	Whole farm	Agroecosystem, pasture	Forage production, herd structure	Sensitivity analysis	n/a
[30]	Australia	Biophysical	Dynamic, stochastic	Herd	Animal health	Herd structure, disease dynamics, animal growth	Sensitivity analysis, convergence testing	R
[31]	United States	Biophysical	Other	Herd	Productivity, resource use	Feed intake, animal growth	Comparison with available data	n/a
[78]	United States	Bioeconomic	Empirical	Herd	Farm management	Herd structure, economic output	n/a	n/a
[32]	Australia	Biological	Other	Whole farm	Productivity, resource use	Animal growth, nutrient requirements	n/a	n/a
[67]	Canada	Bioeconomic	Dynamic, deterministic	Enterprise	Animal production, economics	Economic output	n/a	Vensim
[33]	France	Biophysical	Other	Animal	Energy requirements	Animal growth, nutrient/energy requirements	Sensitivity analysis, comparison to available data	n/a
[34]	Australia	Bioeconomic	Deterministic	Whole farm	Pasture, farm management, economy	Herd structure, animal growth, pasture availability, economic output	Sensitivity analysis, comparison to available data	n/a
[93]	Peru	Biophysical	Dynamic	Whole farm	Economy, forage, productivity	System dynamics, forage production, economic output	n/a	Microsoft Excel, Java
[87]	Japan	Bioeconomic	Other	Whole farm	Animal production, economy, resource use	Nutrient requirement, economic output, GHG emission	Sensitivity analysis,	n/a
[68]	United States	Bioeconomic	Dynamic	Whole farm	Productivity, economy	Biological and economic efficacy, herd structure	Sensitivity analysis,	STELLA
[111]	United States	Biophysical	Other	Whole farm	Productivity, forage	Animal production, forage production	Comparison with available data	n/a
[61]	United States	Population	Deterministic	Enterprise	Environment, resource use	Herd structure	n/a	n/a
[112]	United States	Bioeconomic	Stochastic	Enterprise	Economy	Nutrient requirement, economic output	n/a	n/a
[73]	Australia	Bioeconomic	Static, dynamic	Whole farm	Pasture, economy	Herd structure, economic output,	n/a	Exsys
[113]	United States	Biophysical	Other	Enterprise	Animal production	Forage production, nutrient requirements	n/a	n/a
[88]	Australia	Biophysical	Other	Whole farm	Pasture	Pasture production	Sensitivity analysis	n/a
[114]	Brazil	Bioeconomic	Deterministic, static	Herd	Productivity, economy	Economic output	n/a	Microsoft Excel
[115]	Japan	Bioeconomic	Deterministic	Cow–calf	Economic, productivity	Genetic potential, economic output	n/a	ZPLAN
[35]	United Kingdom	Bioeconomic	Static, deterministic	Whole farm	Economic,	Herd structure, growth, nutrient requirement, economic output	Face validity	n/a
[36]	Canada	Bioeconomic	Deterministic	Herd	Economic, genetic improvement	Economic output, animal growth, nutrient requirements reproductive performance	Comparison to available data	n/a
[97]	United States	Biophysical	Other	Enterprise	Animal production, economy,	Herd structure, economic output	n/a	SAS
[116]	Brazil	Biophysical	Other	Whole farm	Productivity	Animal production	n/a	n/a
[21]	United States	Biophysical	Stochastic	Cow–calf	Environment	Herd structure, nutrient requirements, nutrient availability, reproductive performance	Sensitivity analysis,	R
[62]	France	Population	Dynamic	Herd	Animal production, environment	Herd structure	Sensitivity analysis	n/a
[71]	United States	Biophysical	Dynamic	Whole farm	Pasture	Pasture growth, nutrient intake	n/a	FORTRAN
[22]	Brazil	Bioeconomic	Deterministic, stochastic, empirical	Whole farm	Economy	Animal growth, economic output, pasture production, nutrient requirements	Sensitivity analysis,	Microsoft Excel, @Risk
[92]	Brazil	Biophysical	Other	Cow–calf	Productivity	Herd structure, energy requirements	Comparison to other models	n/a
[117]	Spain	Bioeconomic	Deterministic	Whole farm	Economic, productivity	Economic output	n/a	n/a
[72]	Australia	Agroecosystem	Dynamic	Whole farm	Pasture	Grazing effects, pasture production	n/a	n/a
[59]	Ireland	Biological	Dynamic, deterministic	Herd	Animal reproduction	Herd structure, reproductive performance	Sensitivity analysis	STELLA
[23]	Argentina	Biophysical	Stochastic, dynamic, mechanistic	Whole farm	Farm management	Herbage growth, productivity outcomes	n/a	Java, Oracle
[118]	South Africa	Bioeconomic	Other	Enterprise	Reproduction	Reproductive outcome, economic outcome	n/a	n/a
[119]	France	Biophysical	Dynamic	Whole farm	Management	Herd structure, herbage production, management decisions	Sensitivity analysis	C++
[120]	Brazil	Agroecosystem	Deterministic	Whole farm	Environment	Herd structure, GHG emission	n/a	n/a
[37]	Australia	Biophysical	Other	Whole farm	Productivity, economy	Economic output, animal growth, forage production	n/a	Microsoft Excel
[38]	Australia	Biophysical	Other	Whole farm	Pasture	Pasture growth, animal growth, reproductive output	n/a	n/a
[69]	Australia	Bioeconomic	Dynamic	Enterprise	Productivity, economy	Economic output, animal production	n/a	n/a
[79]	United States	Bioeconomic	Empirical	Cow–calf	Economic, management	Nutrient requirement, herd structure	n/a	n/a
[39]	United States	Biophysical	Dynamic	Cow–calf	Resource use	Herd structure, animal growth, nutrient requirements, forage production, management decisions	Sensitivity analysis	n/a
[75]	Brazil	Bioeconomic	Stochastic	Whole farm	Economics, management	Herd structure, feed production, economic output	n/a	n/a
[121]	France	Bioeconomic	Dynamic	Whole farm	Productivity, economy	Herd structure, feed production, economic output	Comparison to available data	n/a
[40]	Canada	Biophysical	Deterministic	Herd	Productivity,	Feed intake, animal growth, production outcome	Comparison to other models, comparison to available data, sensitivity analysis	n/a
[122]	Argentina	Biophysical	Deterministic	Enterprise	Management, production	Herd structure, pasture production, feed intake	Sensitivity analysis, face validity,	Powersim Studio 8
[41]	United States	Biological	Deterministic	Animal	Net energy required, growth	Energy requirements, animal growth	n/a	n/a
[123]	United States	Biophysical	Stochastic	Whole farm	Animal health, economic	Herd structure, animal disease	Sensitivity analysis	n/a
[124]	Brazil	Bioeconomic	Other	Cow–calf	Economic	Animal production, economic output	n/a	Microsoft Excel
[125]	Nigeria	Bioeconomic	Dynamic	Enterprise	Management	Herd structure, pasture production, economic output	n/a	STELLA
[42]	Vietnam	Biological	Deterministic	Enterprise	Environment	GHG emission, animal growth, energy requirements	Sensitivity analysis	R
[43]	Japan	Bioeconomic	Deterministic	Herd	Management, economy, productivity	Animal growth, herd structure, reproductive performance, production outcome	n/a	n/a
[126]	Uganda	Bioeconomic	Deterministic, stochastic	Herd	Animal health	Herd structure, disease transmission	Compared results with different inputs (rationalism method)	n/a
[83]	Canada	Bioeconomic	Dynamic, deterministic	Whole farm	Economy, management	Herd structure, nutrient requirements, forage production, economic output	Comparison to other models, sensitivity analysis	STELLA
[127]	Uruguay	Bioeconomic	Deterministic	Whole farm	Reproduction, economy,	Reproductive outcome, economic output, feed requirement	n/a	Microsoft Excel
[44]	Colombia	Biophysical	Dynamic, mechanistic	Whole farm	Environment	Animal growth, GHG emission	n/a	Microsoft Excel
[128]	New Zealand	Bioeconomic	Dynamic	Whole farm	Economy	Economic outcome	n/a	n/a
[129]	Argentina	Biophysical	Dynamic	Whole farm	Farm management	Herd structure	n/a	Java
[130]	Canada	Biophysical	Dynamic, stochastic	Herd	Animal health	Herd structure, disease dynamics	n/a	R
[131]	United States	Biophysical	Other	Whole farm	Environment	GHG emission, resource use, economic outcome	Sensitivity analysis	n/a
[45]	Norway	Biophysical	Empirical	Whole farm	Environment	GHG emission, animal growth, herd structure, energy requirement	Sensitivity analysis	Microsoft Excel
[132]	United States	Biophysical	Deterministic	Whole farm	Production, management	Genetic potential, forage intake, herd structure	Comparison to available data	FORTRAN
[84]	Brazil	Bioeconomic	Deterministic, dynamic	Cow–calf	Management, economy, productivity	Herd structure, energy requirement, forage production, economic output	Comparison to other models	Microsoft Excel
[133]	United States	Biophysical	Other	Whole farm	Management	Management decisions, whole-farm scenario	n/a	FORTRAN, BASIC, C++
[24]	United States	Biophysical	Deterministic, dynamic	Herd	Productivity	Herd structure, reproductive performance	n/a	STELLA
[134]	Brazil	Bioeconomic	Dynamic	Herd	Productivity, environment	Herd structure, economic output, land use, GHG emission	n/a	n/a
[46]	Brazil	Bioeconomic	Conceptual	Enterprise	Farm management, economy	Economic output, pasture production, reproductive output, animal growth	Face validity, conceptual validation	n/a
[47]	United States	Biological	Deterministic, mechanistic	Animal	Productivity	Animal growth, forage intake	Comparison to available data	n/a
[48]	United States	Biophysical	Deterministic, stochastic,	Enterprise	Productivity	Forage intake, energy requirement, animal growth, reproductive output	n/a	n/a
[60]	Brazil	Biological	Dynamic	Animal	Management	Metabolic output	n/a	MATLAB
[94]	Kenya	Bioeconomic	Other	Whole farm	Productivity, economy	Economic outcome	n/a	FORTRAN
[98]	United States	Bioeconomic	Dynamic	Enterprise	Economy	Economic output, herd structure	n/a	n/a
[135]	United States	Biophysical	Dynamic	Cow–calf	Economy, management,	Economic output, herd structure	Sensitivity analysis, comparison to available data	STELLA
[49]	Netherlands	Biophysical	Deterministic, mechanistic, dynamic	Herd	Productivity, resource use	Animal growth, forage intake, herd structure, animal production	n/a	R
[63]	France	Population	Stochastic	Cow–calf	Management	Herd structure	Face validity	n/a
[136]	Spain	Biophysical	Dynamic, stochastic	Herd	Productivity	Animal production, reproductive performance, energy requirement	Comparison to available data, sensitivity analysis	n/a
[50]	Spain	Biophysical	Dynamic, stochastic	Herd	Management	Herd structure, animal growth, nutrient requirement, reproductive performance	n/a	Visual Basic
[76]	United States	Bioeconomic	Stochastic, dynamic, deterministic	Herd	Productivity, management, economy	Reproductive performance, economic output, herd structure, feed requirement	n/a	LOTUS 1-2-3
[137]	United States	Bioeconomic	Other	Whole farm	Pasture, management	Pasture production, herd dynamics, economic output, GHG emission	Comparison to available data	n/a
[138]	Czech Republic	Bioeconomic	Dynamic	Enterprise	Management, economy	Herd structure, economic efficacy	n/a	n/a
[139]	Czech Republic	Bioeconomic	Deterministic	Enterprise	Economy, management	Animal production, economic output	n/a	n/a
[140]	United Kingdom	Biophysical	Mechanistic	Whole farm	GHG emission, management	Feed requirement, environmental outcome	n/a	n/a
